# Traumatic testicular dislocation: A case report and literature review

**DOI:** 10.1097/MD.0000000000029137

**Published:** 2022-05-13

**Authors:** Yi-Chen Chiu, Yen-Ko Lin

**Affiliations:** a Department of Emergency Medicine, Kaohsiung Medical University Hospital, Kaohsiung Medical University, Kaohsiung, Taiwan; b Division of Trauma and Surgical Critical Care, Department of Surgery, Kaohsiung Medical University Hospital, Kaohsiung Medical University, Kaohsiung, Taiwan; c Department of Medical Humanities and Education, College of Medicine, Kaohsiung Medical University, Kaohsiung, Taiwan.

**Keywords:** case report, emergent medicine, motorcycle accident, traumatic testicular dislocation

## Abstract

**Introduction:**

Traumatic testicular dislocation is an uncommon complication of blunt scrotal injury and is easily overlooked because of the presence of other severe accompanying injuries. In most cases, an operation is needed for the prevention of malignant change or infertility.

**Patient concerns and diagnosis:**

We report a case of traumatic testicular dislocation with pelvic fracture and internal bleeding in a 27-year-old male with testis rupture after a motorcycle collision.

**Interventions and outcomes:**

He received emergent right radical orchiectomy, and a series of operations for femoral and pelvic fractures were performed after his condition stabilized in the intensive care unit. After 1 month postsurgery, no obvious genitourinary complications were noted.

**Conclusion:**

We suggest scrotum examination in all trauma patients, particularly if a pelvic injury is suspected or in case of a high risk of a motorcycle collision, to avoid missing the diagnosis and prevent severe complications

## 1 Introduction

Testicular dislocation is defined as the testis outside the scrotum, and the first case was reported by Claubry in 1818.^[1]^ It is a rare event with fewer than 200 reported cases.^[2,3]^ Testicular dislocation is most commonly reported due to motorcycle injury (80%) at a young age (in the 20s) and is usually unilateral^[4]^; other causes include falling, explosion to hit during sexual activity, etc. We report a case of traumatic testicular dislocation in a 27-year-old male with testis rupture after a motorcycle collision, along with a brief literature review.

## 2 Case presentation

**Figure 1:**
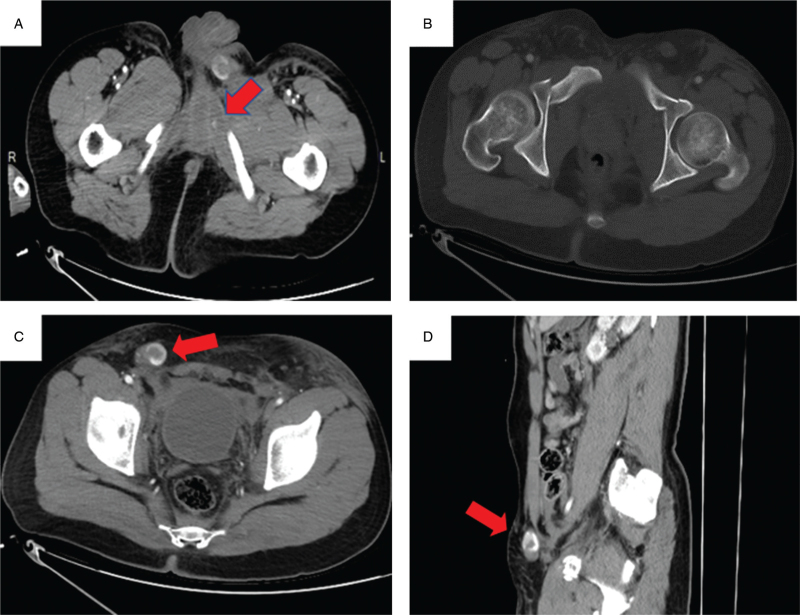
A: CT imaging showing bleeding in left levator ani. B: CT imaging showing bilateral pubic superior and inferior ramus fractures. C: CT imaging showing right testicular dislocation(axial view). D: CT imaging showing right testicular dislocation (sagittal view). CT = computed tomography.

**Figure 2:**
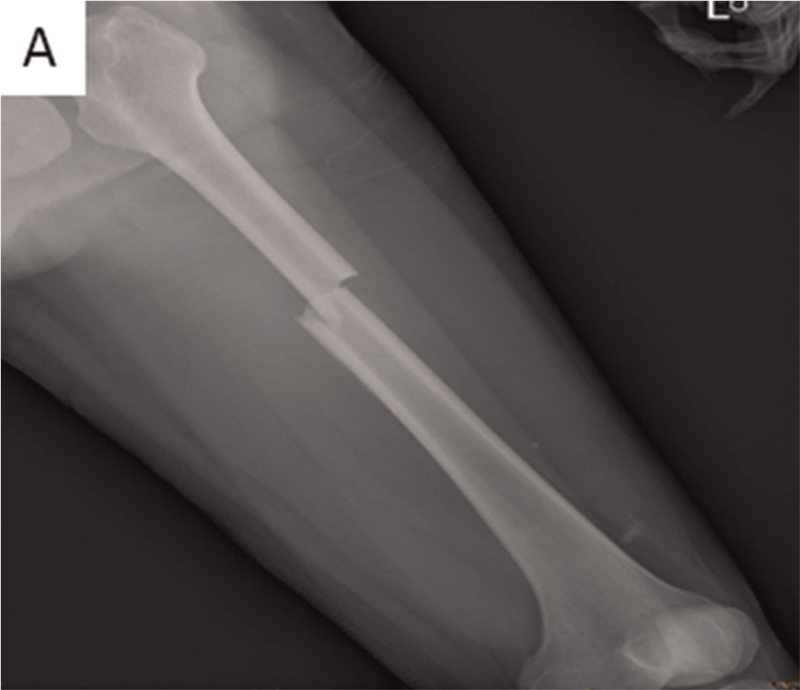
Radiograph showing left femoral shaft fracture.

**Figure 3:**
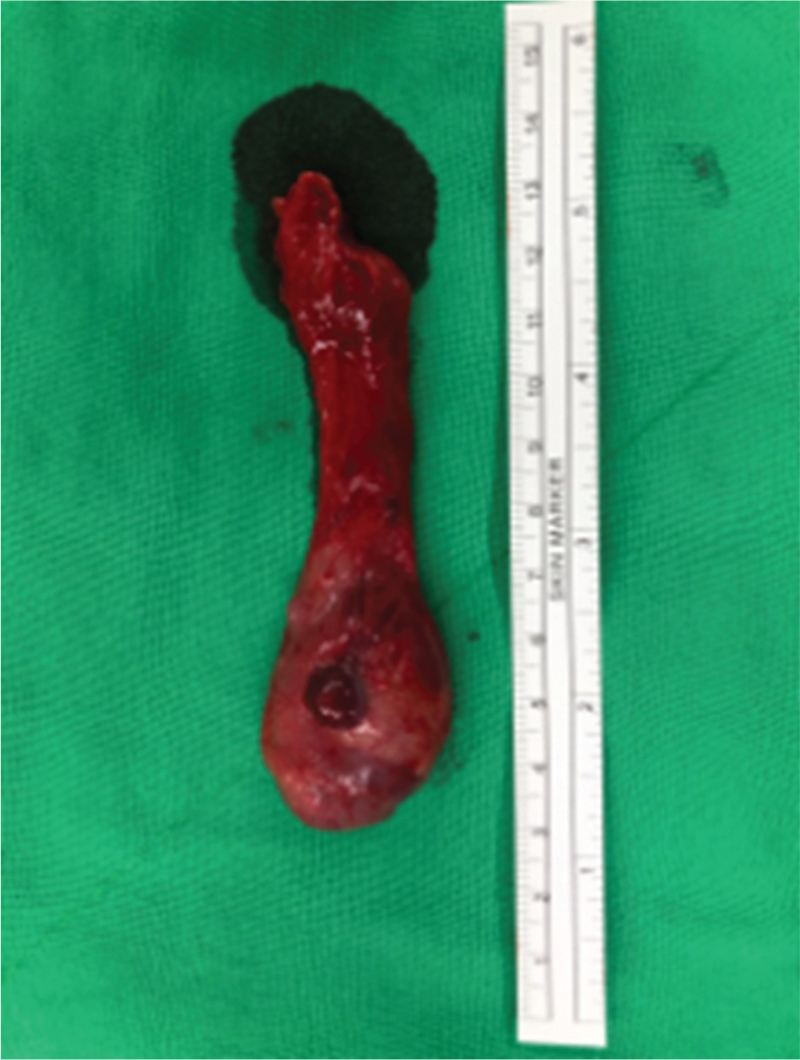
Specimen of right radical orchiectomy.

**Figure 4:**
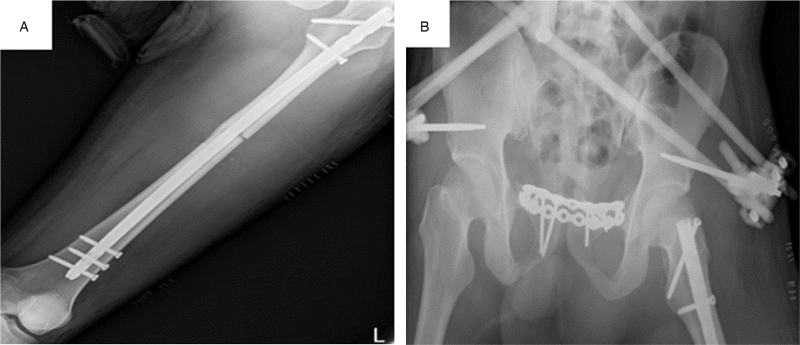
A: Radiograph showing fixation for femoral fractures. B: Radiograph showing fixation for pelvic fractures.

A 27-year-old male was sent to the emergency department in a clinically unstable state after a motorcycle collision. Physical examination (PE) showed multiple contusions and bruises over bilateral lower extremities. A whole-body computer tomography (CT) scan and series radiography of extremities were performed. The findings revealed bilateral pubic superior and inferior ramus fractures with associated bleeding in left levator ani through contrast extravasation, suspected right testicular dislocation, left femoral shaft fracture, and right pneumothorax (Figs. 1 and 2). His Injury Severity Score was 27. Emergency blood transfusion (4 units of packed RBC), resuscitation, chest tube insertion, and angiography were performed. Angiography showed active bleeding from the distal branches of the bilateral internal pudendal arteries, and hence, embolization was performed. After vital sign stabilization, a urologist and an orthopedic surgeon were consulted. Thereafter, emergency surgery was arranged for right testicular dislocation with right testis rupture, and right radical orchiectomy was performed (Fig. 3). The patient was admitted to the intensive care unit after surgery, and a series of operations for femoral and pelvic fractures were performed after his condition stabilized (Fig. 4). After 1 month postsurgery, no obvious genitourinary complications were noted.

## 3 Discussion

Testicular dislocation is easily overlooked because other severe injuries, such as pelvic fracture, long bone fracture, or intra-abdominal bleeding, can accompany at the same time. Although testicular dislocation is usually not fatal, a series of complications, including pain, malignant change, infertility, or abscess/necrosis, may occur if left untreated.^[3,6,7]^ However, the prognosis is often good if treated appropriately. The diagnosis might be missed in patients with nonsevere trauma where CT is not required or if the scrotum remains unexamined. Some studies reported nine groin trauma patients where the testicular dislocation diagnosis was initially missed, but the delayed diagnosis was made within an average of 19 days.^[8]^ For early diagnosis of testicular dislocation, PE and understanding the cause of injury are important, especially in cases involving palpation of the scrotum and presentation of scrotal hematoma.^[3]^ Recently, CT has also been reported to be a good tool, and the findings play a critical role in the testicular dislocation diagnosis in many trauma patients.^[9]^ Some authors have recommended CT to be more sensitive in detecting testicular dislocation,^[8,9]^ particularly in cases involving hollow viscus injury, pancreatic injury, and pelvic injury.^[8]^

Missed injury is not uncommon in patients with major trauma in the emergency department, accounting for approximately 12.1% of cases, especially in younger patients with more severe injuries and polytrauma.^[10,11]^ Additionally, the pelvis has the highest incidence rate of missed injury, and clinically significant missed injury (defined as the Abbreviated Injury Scale ≥ 2) is significantly associated with pelvic injury (hazard ratio, 2.19).^[10]^

Some cases of testicular dislocation are treated with conservative treatment (e.g., observation or manual reduction);^[7,12]^ however, surgical orchidopexy is needed in most cases and is recommended as the preferred initial treatment due to failure of closed reduction, the possibility of torsion, or difficulty to locate the ruptured testis.^[6]^

We searched PubMed and reviewed some case reports or case series with the keyword “testicular dislocation” from January 1965 until August 2021(Table 1). Initially, 110 articles were identified, and 12 articles not published in English were excluded. Rest 98 articles were retrieved for review, and 53 articles not meeting the interest or lacking full text were excluded. Finally, 45 reports containing 105 cases published until August 2021 were analyzed. The most common cause of testicular dislocation in these reports is motorcycle accidents (80%), followed by traffic and road accidents (5.7%) and blunt/hit injuries (3.8%). The most commonly used diagnostic method is PE (34.2%), followed by sonography (21.9%) and CT (19%). However, the values changed to PE (53.7%), sonography (34.3%), and CT (29.9%) if the numbers of patients without diagnostic methods were excluded. These results are consistent with the current published reports and suggest motorcycle accidents as the most common cause for testicular dislocation and PE as the most important diagnostic method.

**Table 1:** Case reports and series of testicular dislocation.

Report	Authors	Number of patients	Mechanism	Diagnostic method
1	Claubry ^[1]^	1	Wagon wheel trauma	Not mentioned
2	Morgan ^[13]^	4	2 Road accidents1 MA1 Bicycle accident	PE, OP
3	Edson and Meek ^[14]^	1	Straddle injury	PE
4	Kauder and Bucchiere ^[15]^	1	MA	PE
5	Nagarajan et al ^[16]^	3	3 MAs	PE
6	Masui et al ^[17]^	1	MA	Not mentioned
7	Feder et al ^[18]^	1	Hit by knee on scrotum	PE
8	Lee et al ^[19]^	2	1 MA1 Automotive accident	PE
9	Madden ^[7]^	1	MA	PE
10	Schwartz and Faerber ^[20]^	1	Pedestrian-motor vehicleaccident	PE, US
11	Toranji and Barbaric^[21]^	1	MA	CT
12	O’Donnell et al^[22]^	3	MA	PE
13	Shefi S et al. ^[23]^	1	MA	PE, US, CT
14	Kochakarn et al ^[24]^	36	MA	Not mentioned
15	Lo’pez Alcina et al^[2]^	2	1 MA1 Kick	PE, US
16	Tsai et al^[25]^	1	MA	US, CT
17	Bromberg et al^[5]^	1	MA	CT
18	O’Brien et al^[26]^	1	MA	US
19	Ko et al^[8]^	9	7 MAs1 Explosive injury1 Seat belt injury	CT(7), US(2)
20	Bedir et al^[27]^	1	MA	US, MRI
21	Luján Marco et al^[28]^	1	MA	CT
22	Ihama et al^[29]^	1	MA	Autopsy
23	Sakamoto et al^[30]^	1	MA	PE, US, MRI
24	Aslam et al^[31]^	1	Blunt injury to scrotum	PE, US
25	Ezra et al^[9]^	1	MA	CT
26	Vasudeva et al^[32]^	1	MA	US
27	Perera et al^[33]^	1	MA	PE, US, CT
28	Jecmenica et al^[34]^	2	MA	Autopsy
29	Tsurukiri et al^[35]^	1	MA	PE, CT
30	Naseer et al^[36]^	1	Traffic accident	PE, CT
31	Boudissa et al^[37]^	1	MA	CT
32	Matzek and Linklater ^[38]^	1	Blunt abdominopelvic injury	PE, US
33	Meena et al^[39]^	1	MA	PE, US
34	Zavras et al^[40]^	1	Falling astride on a crossbar	PE, US
35	Gómez et al^[41]^	7	6 MAs1 pelvic crush injury	PE, US, autopsy(2)
36	Pesch and Bradin ^[42]^	1	Straddle injury	US
37	Wiznia et al^[43]^	1	MA	PE, US
38	Kim et al^[44]^	1	construction accident	CT
39	de Carvalho et al^[6]^	1	MA	PE, US
40	Shirono et al^[12]^	1	Falling down	US, MRI
41	Middleton et al^[45]^	2	MA	CT
42	Riaza Montes et al^[3]^	1	Uncertain (alcohol abuse)	PE, US
43	Bernhard et al^[46]^	1	MA	During OP
44	Mangual-Perez et al^[47]^	1	MA	During OP
45	Naik et al^[48]^	1	MA	PE

CT = computed tomography, MA = motorcycle accident, OP = operation, PE = physical examination, US = ultrasonography.

In the present case, the scrotum was not examined initially because of other severe injuries and unstable vital signs. Therefore, the diagnosis of traumatic testicular dislocation was missed, though it was finally detected using CT. Thus, we suggest scrotum examination in all trauma patients, particularly if a pelvic injury is suspected or in case of a high risk of a motorcycle collision, to avoid missing the diagnosis and prevent severe complications.

## Acknowledgments

We thank Dr Chen and Dr Lin for performing the surgeries.

## Author contributions

**Writing – original draft:** Yi-Chen Chiu.

**Writing – review & editing:** Yen-Ko Lin.

## References

[1] Claubry E. Observation sur une rétrocession subite des deux testicules dans l’abdomen, à suite d’une violente compression de la partie inférieure de la paroi abdominale par une roue de charette. J Gen Med Chir Pharm 1818;64:325–30.

[2] López Alcina E Martín JC Fuster A Pérez J Puertas M Moreno J. Testicular dislocation. Report of 2 new cases and review of the literature. Actas Urol Esp 2001;25:299–302. 11455833 10.1016/s0210-4806(01)72619-8

[3] Riaza Montes M Palacios Ramos J Gallego Sánchez JA. Testicular dislocation: an atypical case and review of the literature. Urol Case Rep 2020;33:101405. 33102103 10.1016/j.eucr.2020.101405 PMC7574044

[4] Lenfant M Escoffier A Chevallier O. Traumatic ectopic dislocation of testis: an easily overlooked occurrence of blunt injury in polytrauma patients. Quant Imaging Med Surg 2019;9:2008–11. 31929975 10.21037/qims.2019.11.11 PMC6942966

[5] Bromberg W Wong C Kurek S Salim A. Traumatic bilateral testicular dislocation. J Trauma 2003;54:1009–11. 12777919 10.1097/01.TA.0000055220.78753.25

[6] de Carvalho NMN Marques ACX de Souza IT. Bilateral traumatic testicular dislocation. Case Rep Urol 2018;2018:7162351. 29862114 10.1155/2018/7162351 PMC5971239

[7] Madden JF. Closed reduction of a traumatically dislocated testicle. Acad Emerg 1994;1:272–5. 10.1111/j.1553-2712.1994.tb02444.x 7621208

[8] Ko SF Ng SH Wan YL. Testicular dislocation: an uncommon and easily overlooked complication of blunt abdominal trauma. Ann Emerg Med 2004;43:371–5. 14985665 10.1016/S0196064403007492

[9] Ezra N Afari A Wong J. Pelvic and scrotal trauma: CT and triage of patients. Abdom Imaging 2009;34:541–4. 18543018 10.1007/s00261-008-9417-3

[10] Chen CW Chu CM Yu WY Lou YT Lin MR. Incidence rate and risk factors of missed injuries in major trauma patients. Accid Anal Prev 2011;43:823–8. 21376872 10.1016/j.aap.2010.11.001

[11] Lin YK Lin CJ Chan HM. Surgeon commitment to trauma care decreases missed injuries. Injury 2014;45:83–7. 23131679 10.1016/j.injury.2012.10.019

[12] Shirono Y Yamaguchi S Takahashi E Terunuma M. Conservative management of bilateral traumatic testicular dislocation in a 10-year-old boy. J Rural Med 2018;13:82–5. 29875902 10.2185/jrm.2952 PMC5981024

[13] Morgan A. Traumatic luxation of the testis. Br J Surg 1965;52:669–72. 14338314 10.1002/bjs.1800520908

[14] Edson M Meek JM. Bilateral testicular dislocation with unilateral rupture. J Urol 1979;122:419–20. 470026 10.1016/s0022-5347(17)56441-1

[15] Kauder DH Bucchiere JJ Jr. Bilateral traumatic testicular dislocation. J Urol 1980;123:606. 10.1016/s0022-5347(17)56054-1 7365915

[16] Nagarajan VP Pranikoff K Imahori SC Rabinowitz R. Traumatic dislocation of testis. Urology 1983;22:521–4. 6649208 10.1016/0090-4295(83)90233-9

[17] Masui Y Ueda K Ootaguro K. Traumatic dislocation of the testis. A case report. Hinyokika Kiyo 1989;35:1417–20. 2683654

[18] Feder M Sacchetti A Myrick S. Testicular dislocation following minor scrotal trauma. Am J Emerg Med 1991;9:40–2. 1985649 10.1016/0735-6757(91)90012-9

[19] Lee JY Cass AS Streitz JM. Traumatic dislocation of testes and bladder rupture. Urology 1992;40:506–8. 1466102 10.1016/0090-4295(92)90403-j

[20] Schwartz SL Faerber GJ. Dislocation of the testis as a delayed presentation of scrotal trauma. Urology 1994;43:743–5. 8165779 10.1016/0090-4295(94)90203-8

[21] Toranji S Barbaric Z. Testicular dislocation. Abdom Imaging 1994;19:379–80. 8075571 10.1007/BF00198205

[22] O’Donnell C Kumar U Kiely EA. Testicular dislocation after scrotal trauma. Br J Urol 1998;82:768. 9839604 10.1046/j.1464-410x.1998.00792.x

[23] Shefi S Mor Y Dotan ZA Ramon J. Traumatic testicular dislocation: a case report and review of published reports. Urology 1999;54:744. 10.1016/s0090-4295(99)00238-1 10754145

[24] Kochakarn W Choonhaklai V Hotrapawanond P Muangman V. Traumatic testicular dislocation a review of 36 cases. J Med Assoc Thai 2000;83:208–12. 10710892

[25] Tsai HN Wu WJ Huang SP. Bilateral traumatic testicular dislocation--a case report. Kaohsiung J Med Sci 2002;18:95–8. 12056175

[26] O’Brien MF Collins DA McElwain JP Akhtar M Thornhill JA. Traumatic retrovesical testicular dislocation. J Urol 2004;171:798. 14713818 10.1097/01.ju.0000106363.44048.98

[27] Bedir S Yildirim I Sümer F Tahmaz L Dayanç M Peker AF. Testicular dislocation as a delayed presentation of scrotal trauma. J Trauma 2005;58:404–5. 15706214 10.1097/01.ta.0000075853.25824.c5

[28] Luján Marco S Budía Alba A Bango García V Ramirez Backhaus M Delgado Oliva FJ Jiménez Cruz JF. Traumatic testicular dislocation. Actas Urol Esp 2006;30:409–11. 16838614 10.1016/s0210-4806(06)73466-0

[29] Ihama Y Fuke C Miyazaki T. A two-rider motorcycle accident involving injuries around groin area in both the driver and the passenger. Leg Med (Tokyo) 2007;9:274–7. 17562381 10.1016/j.legalmed.2007.03.003

[30] Sakamoto H Iwasaki S Kushima M Shichijo T Ogawa Y. Traumatic bilateral testicular dislocation: a recovery of spermatogenesis by orchiopexy 15 years after the onset. Fertil Steril 2008;90: 2009.e9-11. 10.1016/j.fertnstert.2008.01.105 18541235

[31] Aslam MZ Thwaini A Sundaram SK. Testicular dislocation: a rare consequence of blunt scrotal injury. Can Urol Assoc J 2009;3:E1–3. 19543451 10.5489/cuaj.1085 PMC2692158

[32] Vasudeva P Dalela D Singh D Goel A. Traumatic testicular dislocation: a reminder for the unwary. J Emerg Trauma Shock 2010;3:418–9. 21063572 10.4103/0974-2700.70762 PMC2966582

[33] Perera E Bhatt S Dogra VS. Traumatic ectopic dislocation of testis. J Clin Imaging Sci 2011;1:17. 21966614 10.4103/2156-7514.77124 PMC3177419

[34] Ječmenica DS Alempijević DM Pavlekić S Aleksandrić BV. Traumatic testicular displacement in motorcycle drivers. J Forensic Sci 2011;56:541–3. 21306375 10.1111/j.1556-4029.2010.01682.x

[35] Tsurukiri J Kaneko N Mishima S. Bilateral traumatic testicular dislocation. Urology 2011;78:1306. 21458036 10.1016/j.urology.2011.01.052

[36] Naseer A King D Lee H Vale J. Testicular dislocation: the importance of scrotal examination in a trauma patient. Ann R Coll Surg Engl 2012;94:e109–10. 22391381 10.1308/003588412X13171221502266 PMC5827246

[37] Boudissa M Ruatti S Maisse N. Bilateral testicular dislocation with pelvic ring fracture: a case report and literature review. Orthop Traumatol Surg Res 2013;99:485–7. 23648314 10.1016/j.otsr.2013.01.010

[38] Matzek BA Linklater DR. Traumatic testicular dislocation after minor trauma in a pediatric patient. J Emerg Med 2013;45:537–40. 23899815 10.1016/j.jemermed.2012.11.093

[39] Meena S Barwar N Chowdhury B. Double trouble: testicular dislocation associated with hip dislocation. J Emerg Trauma Shock 2014;7:58–9. 24550637 10.4103/0974-2700.125646 PMC3912658

[40] Zavras N Siatelis A Misiakos E Bagias G Papachristos V Machairas A. Testicular dislocation after scrotal trauma: a case report and brief literature review. Urol Case Rep 2014;2:101–4. 26955557 10.1016/j.eucr.2014.02.004 PMC4733017

[41] Gómez RG Storme O Catalán G Marchetti P Djordjevic M. Traumatic testicular dislocation. Int Urol Nephrol 2014;46:1883–7. 24869967 10.1007/s11255-014-0736-8

[42] Pesch MH Bradin S. The case of the missing testicle: blunt scrotal trauma in the pediatric emergency department. Pediatr Emerg Care 2014;30:824–5. 25373570 10.1097/PEC.0000000000000270

[43] Wiznia DH Wang M Yeon-Kim C Tomaszewski P Leslie MP. Traumatic testicular dislocation associated with lateral compression pelvic ring injury and T-shaped acetabulum fracture. Case Rep Orthop 2016;2016:9706392. 27672464 10.1155/2016/9706392 PMC5031850

[44] Kim WY Lee SW Jang H Kim DY. Delayed detection of testicular dislocation with pelvic ring fracture: a case report. J Orthop Sci 2016;21:702–4. 26740454 10.1016/j.jos.2015.06.024

[45] Middleton AH Martin JM Wittmann TA Schmeling GJ. Testicular dislocation after pelvic ring injury: a report of 2 cases. JBJS Case Connect 2019;9:e0141. 31850961 10.2106/JBJS.CC.19.00141

[46] Bernhard Z Myers D Passias BJ Taylor BC Castaneda J. Testicular dislocation after unstable pelvic ring injury. Cureus 2021;13:e13119. 33728137 10.7759/cureus.13119 PMC7935200

[47] Mangual-Perez D Torres-Cintron C Colon-Morillo R Lojo-Sojo L Puras-Baez A. Blunt abdominopelvic trauma complicated by traumatic testicular dislocation in a 19-year-old male patient: a case report. JBJS Case Connect 2021;11. 10.2106/JBJS.CC.20.00911 34293774

[48] Naik MN Shaikh OH Vijayakumar C Kumbhar US. Rare case of traumatic bilateral testicular dislocation. BMJ Case Rep 2021;14:e244085. 10.1136/bcr-2021-244085 PMC8386237 34429293

